# Supplementary material for: Effect of Six Lactic Acid Bacteria Strains on Physicochemical Characteristics, Antioxidant Activities and Sensory Properties of Fermented Orange Juices
Source: Foods. 2022 Jun 28;11(13):1920. doi: 10.3390/foods11131920 (PMC9265423; doi:10.3390/foods11131920)
Supplement: Supplementary file 1 [file foods-11-01920-s001.zip › foods-1782667-supplementary.pdf]

Supplementary Materials

Table S1. Pearson correlation coefficients of aroma-active compounds with OAV ≥ 1 and aroma flavors of the fermented orange juices and original orange juice (control).

Compounds	Ethyl acetate	Ethyl butyrate	Ethyl 3-hydroxyhexanoate	*α*-Pinene	*β*-Myrcene	D-Limonene	2-Carene	3-Carene	Terpinolene	*α*-Terpinene
Ethyl butyrate	-0.407	1								
Ethyl 3-hydroxyhexanoate	0.067	-0.236	1							
α-Pinene	-0.152	0.877**	-0.204	1						
β-Myrcene	-0.003	-0.101	-0.596	0.188	1					
D-Limonene	0.226	-0.771*	-0.200	-0.585	0.647	1				
2-Carene	-0.111	0.273	0.584	0.039	-0.695	-0.547	1			
3-Carene	0.207	0.385	-0.401	0.320	-0.134	-0.493	0.178	1		
Terpinolene	-0.297	-0.068	0.355	-0.287	-0.685	-0.461	0.204	0.168	1	
α-Terpinene	-0.042	0.769*	0.066	0.805*	-0.180	-0.774	0.483	0.630	-0.002	1
p-Cymene	-0.255	0.834*	-0.156	0.564	-0.355	-0.739	0.626	0.528	-0.052	0.708
Valencene	0.284	-0.625	0.510	-0.555	-0.343	0.228	-0.237	-0.543	0.425	-0.605
β-Caryophyllene	0.105	-0.223	0.614	-0.352	-0.755*	-0.402	0.587	0.324	0.762*	0.204
β-Humulene	-0.167	0.898**	0.033	0.884**	-0.238	-0.871*	0.408	0.516	0.054	0.951**
γ-Selinene	0.345	-0.813*	-0.244	-0.744	0.213	0.680	-0.647	-0.149	0.156	-0.805
Acetic acid	-0.339	0.668	-0.319	0.420	-0.104	-0.502	0.545	0.640	-0.094	0.646
Linalool	-0.119	0.822*	-0.035	0.601	-0.538	-0.928**	0.571	0.651	0.222	0.799*
(−)-4-Terpineol	-0.146	0.828*	0.002	0.627	-0.546	-0.959**	0.547	0.645	0.287	0.822*
α-Terpineol	-0.158	0.863*	0.102	0.720	-0.504	-0.965**	0.559	0.544	0.223	0.876*
Citronellol	-0.093	0.750	0.278	0.716	-0.491	-0.942**	0.514	0.491	0.310	0.902*
Ethanol	-0.248	0.540	-0.097	0.304	-0.574	-0.824*	0.158	0.568	0.711	0.438
1-Octanol	-0.379	0.820*	0.051	0.706	-0.355	-0.911**	0.444	0.585	0.371	0.884*
Carvone	-0.278	-0.273	0.343	-0.645	-0.660	-0.042	0.306	-0.383	0.534	-0.532
Nootkatone	-0.406	0.385	0.358	0.381	-0.123	-0.446	0.600	0.282	0.173	0.702
(+)-Nootkatone	0.182	-0.517	0.320	-0.557	-0.433	0.090	-0.276	-0.347	0.603	-0.589
Hexanal	-0.167	0.898**	0.033	0.884**	-0.238	-0.871*	0.408	0.516	0.054	0.951**
Pentanal	-0.167	-0.240	-0.280	-0.104	0.555	0.372	-0.206	0.290	-0.047	0.050
(Z)-citral	-0.279	-0.453	0.809*	-0.462	-0.345	0.087	0.323	-0.493	0.514	-0.227
Aroma flavors	0.182	0.120	0.206	-0.035	-0.742	-0.667	0.322	0.626	0.775*	0.354

Table S1. (*continued*)

Compounds	p-Cym-ene	Valenc-ene	β-Cary-ophylle-ne	β-Hum ulene	γ-Selin-ene	Acetic acid	Linalool	(−)-4-Terpi -neol	α-Terpi-neol	Citronellol
Valencene	-0.694	1								
β-Caryophyllene	0.035	0.282	1							
β-Humulene	0.751	-0.517	0.087	1						
γ-Selinene	-0.771*	0.569	0.011	-0.838*	1					
Acetic acid	0.885**	-0.891**	0.054	0.584	-0.627	1				
Linalool	0.909**	-0.468	0.260	0.859*	-0.691	0.717	1			
(−)-4-Terpineol	0.869*	-0.429	0.295	0.888**	-0.693	0.674	0.994**	1		
α-Terpineol	0.841*	-0.414	0.250	0.947**	-0.791*	0.619	0.965**	0.981**	1	
Citronellol	0.648	-0.268	0.371	0.944**	-0.751	0.438	0.853*	0.896**	0.950**	1
Ethanol	0.473	-0.030	0.407	0.566	-0.192	0.315	0.735	0.777*	0.703	0.666
1-Octanol	0.707	-0.480	0.314	0.921**	-0.728	0.631	0.846*	0.891**	0.911**	0.920**
Carvone	-0.023	0.509	0.350	-0.410	0.227	-0.160	-0.038	-0.058	-0.133	-0.232
Nootkatone	0.391	-0.510	0.412	0.570	-0.682	0.547	0.369	0.407	0.478	0.576
(+)-Nootkatone	-0.568	0.941**	0.329	-0.465	0.629	-0.759*	-0.309	-0.270	-0.309	-0.217
Hexanal	0.751	-0.517	0.087	1.000**	-0.838*	0.584	0.859*	0.888**	0.947**	0.944**
Pentanal	-0.255	-0.416	0.069	-0.167	0.197	0.204	-0.338	-0.317	-0.339	-0.248
(Z)-citral	-0.428	0.497	0.580	-0.279	0.048	-0.371	-0.366	-0.312	-0.248	-0.057
Aroma flavors	0.248	0.202	0.785*	0.348	0.024	0.138	0.577	0.613	0.527	0.573

Table S1. (*continued*)

Compounds	Ethanol	1-Octanol	Carvone	Nootka-t one	(+)-Nootka tone	Hexanal	Pentanal	(Z)-citral	Aroma flavors
1-Octanol	0.735	1							
Carvone	0.191	-0.234	1						
Nootkatone	0.12	0.682	-0.294	1					
(+)-Nootkatone	0.242	-0.354	0.606	-0.571	1				
Hexanal	0.566	0.921**	-0.41	0.57	-0.465	1			
Pentanal	-0.248	0.007	-0.474	0.457	-0.422	-0.167	1		
(Z)-citral	-0.16	-0.083	0.421	0.395	0.364	-0.279	0.146	1	
Aroma flavors	0.817*	0.52	0.225	0.126	0.395	0.348	-0.166	0.065	1

*Correlation is significant at $p<0.05$.**Correlation is extremely significant at $p<0.01$.

Table S2. Pearson correlation coefficients of the physicochemical characteristics, viable counts and taste flavors of the fermented orange juices and original orange juice (control).

	Viable bacterias	Soluble sugars	TA	SSC	pH	SSC/TA ratio	Organic acids	Vc	Total flavones	Limo-nin	Sweet-ness	Sour-ness
Viable bacterias	1											
Soluble sugars	-0.949**	1										
TA	0.548	-0.335	1									
SSC	-0.955**	0.961**	-0.509	1								
pH	-0.954**	0.906**	-0.532	0.922**	1							
SSC/TA ratio	-0.890*	0.760*	-0.863*	0.864*	0.863*	1						
Organic acids	0.869*	-0.690	0.865*	-0.792*	-0.852*	-0.973**	1					
Vc	0.255	-0.430	-0.597	-0.216	-0.162	0.177	-0.221	1				
Total flavones	-0.574	0.762*	0.301	0.593	0.593	0.194	-0.144	-0.733	1			
Limonin	-0.747	0.808*	-0.151	0.682	0.654	0.510	-0.505	-0.435	0.766*	1		
Sweetness	-0.659	0.480	-0.781*	0.588	0.767*	0.818*	-0.836*	0.286	0.018	0.127	1	
Sourness	0.917**	-0.800*	0.810*	-0.893**	-0.904**	-0.986**	0.974**	-0.150	-0.288	-0.590	-0.784*	1

SSC/TA ratio: soluble solid content/ total acid content ratio. *Correlation is significant at *p*<0.05.**Correlation is extremely significant at *p*<0.01.
